# Effects of Different Irrigation Solutions on Root Fracture Resistance: An *in Vitro* Study

**DOI:** 10.22037/iej.v13i3.19247

**Published:** 2018

**Authors:** Melissa Cristina Lantigua Domínguez, Victor Feliz Pedrinha, Lorena Cássia Oliveira Athaide da Silva, Mara Eliane Soares Ribeiro, Sandro Cordeiro Loretto, Patrícia de Almeida Rodrigues

**Affiliations:** a *Department of Endodontics, Federal University of Pará, School of Dentistry, Belém, PA, Brazil; *; b *Department of Operative Dentistry, Federal University of Pará, School of Dentistry, Belém, PA, Brazil*

**Keywords:** Chlorhexidine, EDTA, Etidronic Acid, Root Canal Irrigant, Sodium Hypochlorite

## Abstract

**Introduction:**

The aim of this *in vitro* study was to evaluate the effects of sodium hypochlorite (NaOCl), ethylenediaminetetraacetic acid (EDTA), chlorhexidine (CHX) and hydroxyethylidene bisphosphonate (HEBP), also known as etidronate, on susceptibility to root fracture resistance (RFR) in human teeth subjected to endodontic preparation.

**Methods and Materials:**

Seventy extracted single-rooted human teeth were selected, endodontically prepared using the ProTaper Next rotary system (PTN, Dentsply, Maillefer, Ballaigues, Switzerland) and then randomly divided according to the following irrigation regimes (*n*=10): G1, saline solution (0.9% NaCl); G2, 2.5% NaOCl + 17% EDTA; G3, 2% CHX gel + 17% EDTA; and G4, a mixture of 5% NaOCl + 18% HEBP. After this step, all samples received a final irrigation with distilled water. The samples were subjected to axial forces by mechanical compression testing in a universal testing machine (Dynamometers KRATOS, LTDA, SP, Brazil). Data analyses included the Shapiro-Wilk normality test, analysis of variance (one-way ANOVA) and a subsequent multiple comparison test (Tukey’s test).

**Results:**

The results indicated that G1 (0.9% NaCl) presented greater resistance to root fracture. No significant differences were observed in G2 (2.5% NaOCl + 17% EDTA) and G3 (2% CHX gel + 17% EDTA). A significant difference was identified in G4 (mixture of 5% NaOCl + 18% HEBP) (*P*<0.05).

**Conclusion:**

A mixture of 5% NaOCl + 18% HEBP resulted in a lower fracture resistance when used to irrigate canals during endodontic instrumentation.

## Introduction

The clinical success of endodontic treatment relies on the effectiveness of debridement of the root canal system through instrumentation and disinfection as well as the sealing of the previously enlarged endodontic cavity [1]. Because some areas are inaccessible to instruments, thus preventing mechanical cleaning, the use of chemical substances is essential, making it possible to disinfect and remove debris but also working as a lubricant in the root canals, thereby reducing mechanical stress on the endodontic instruments and minimizing their fracture risks [2]. However, although these solutions are important for cleaning and disinfecting the root canal, they are also capable of altering the chemical and structural properties of dentin by modifying the proportion of calcium and phosphate minerals [3, 4]. 

Several irrigation solutions are used in endodontic therapy, such as sodium hypochlorite (NaOCl), chlorhexidine (CHX), ethylenediaminetetraacetic acid (EDTA) and hydroxyethylidene bisphosphonate, also known as etidronate (HEBP) [4, 5]. The mechanical properties of dentin affected by irrigation agents include microhardness, flexural strength, modulus of elasticity [1], permeability and solubility [6]. Relating these properties to the possibility of clinical occurrences, the resistance of root canals to functional loads may decrease, and the roots can become more susceptible to fracture [4, 7]. 

Dentin comprises a complex organic and inorganic structure [8]; the main component of its organic matrix is type I collagen, which is responsible for the hardness of the tissues and is protected by apatite crystals [9]. NaOCl is the most used irrigation agent in endodontic practice and is characterized by a proteolytic action on the tissues, which negatively affects the dentin and causes exhaustion of the organic components [8] in addition to reducing dentin moisture, which is the primary cause attributed to the fragility of a pulped tooth [10]. EDTA is a chelating agent used for the dissolution of inorganic components resulting from the erosion of peritubular and intertubular dentin. When combined with NaOCl, EDTA increases the effectiveness of smear layer removal by demineralizing the inorganic components of dentin through the chelation of calcium ions present in the hydroxyapatite, the main inorganic compound of dentin. This demineralization process was observed in previous studies that used 17% EDTA [4, 11].

The use of HEBP, a biocompatible chelating agent, in combination with NaOCl has been proposed as an alternative irrigation technique for removing the smear layer [12]. This technique is similar to the use of EDTA but yields reduced levels of debris, and the compound provides little interference with the solvent and antimicrobial properties of NaOCl [4, 12, 13]. According to the previous findings, it was believed that a mixture of 5% NaOCl and 18% HEBP would promote a direct action on collagen fibers, resulting in greater tubular opening, and it has been suggested that the use of HEBP may have led to a more superficial action of NaOCl on the organic portion of dentin [4, 12].

Root fracture is a problem with clinical relevance for endodontics because it is associated with a poor prognosis of the affected tooth [14] and is considered the main cause of tooth loss after root canal treatment [15]. *In vitro* fracture resistance experiments aim to provide information and remain important research methods for developing new techniques and materials [16]. Although some studies have evaluated the effect of irrigation solutions on radicular dentin mechanical properties, the contradictory results obtained, reinforce the need for further investigations on this topic [1, 4, 7, 9-13, 17]. Furthermore, to the best of our knowledge, no studies have evaluated the isolated effect of different irrigation regimes with different chelating agents on root fracture resistance (RFR) after instrumentation, while also taking into account the possibility of root fracture occurring between endodontic treatment sessions. Thus, the aim of this *in vitro* study was to assess the fracture resistance of root dentin following the application of different irrigation solutions.

## Materials and Methods

***Solutions***

The substances evaluated in the present study were saline solution (0.9% sodium chloride), 2.5 and 5% NaOCl, 18% HEBP, 17% EDTA and 2% CHX gel. Saline solution (NaCl) was acquired from a pharmacy and was used as a control. 

A stock solution of NaOCl (Sigma-Aldrich, St. Louis, MO, USA) was iodometrically titrated to determine its content of available chlorine. Then, the solution was diluted to 2.5% and 5% NaOCl using distilled water. To obtain solutions of 18% HEBP (Zschimmer & Schwarz Mohsdorf GmbH & Co KG, Burgstadt, Germany), the pure chemical was mixed with deionized water. Also, 17% EDTA was prepared by dissolving disodium EDTA (Sigma-Aldrich, St. Louis, MO, USA) in distilled water with the aid of sodium hydroxide (NaOH) (Sigma-Aldrich, St. Louis, MO, USA); the pH was then adjusted to 7.0 by adding hydrochloric acid (HCl) (Sigma-Aldrich, St. Louis, MO, USA). All chemical substances were prepared and used immediately after mixing. A fresh 1:1 mixture of 5% NaOCl and 18% HEBP was prepared immediately before the experiments, producing a solution that contained 2.5% NaOCl and 9% HEBP as previously described [4, 18]. Then 2% CHX gel was acquired by manipulation in a pharmacy (Personale, Belém, PA, Brazil).

All solutions were stored in dark containers at 5^°^C between experiments in a refrigerator (Consul, São Bernardo do Campo, SP, Brazil). Before use, the substances were removed from the refrigerator and were allowed to equilibrate to room temperature for 60 min.

***Selection and preparation of specimens***

This study was approved by the Research and Ethics Committee of the local university (No.: 086617/2016). Based on clinical indicators, 40 newly extracted human pre-molars with a single canal were selected by periapical radiography. The teeth were collected from adult patients between 30 and 45 years old. Tissues and residues were removed from the root surfaces using Gracey periodontal curettes (SSWhite, Duflex, RJ, Brazil) and were stored in saline solution under refrigeration (5^°^C) for up to 30 days. Teeth that presented immature apexes, root caries, root fractures, cracks, lacerations, sharp curvatures, canal calcifications or endodontic treatment were excluded; these features were identified with the aid of a stereomicroscope (SM X800, Nikon Co., NY, USA) under ×20 magnification.

The teeth were sectioned at the cementoenamel junction at 13±1 mm from the apex using diamond discs (KG Sorensen Ind. and Com., SP, Brazil) mounted on a low-speed motor (Kavo do Brasil, Joinville, Brazil) under copious water irrigation. After sectioning, the standardization of the buccolingual and mesiodistal dimensions of the specimens as well as the dentin thickness of the different faces were determined using an electronic pachymeter (Digital Caliper 150 mm, Digimess, SP, Brazil). This procedure was utilized to obtain roots with similar dentin thickness. The data were analyzed after using the Shapiro-Wilk normality test to verify the distribution of dentin thickness in the groups. Next, the samples were randomly divided into 4 groups according to irrigation solution and the results are presented in Table 1.

The same operator performed all procedures. The apical foramen of each root was sealed with Topdam (FGM, Joinville, SC, Brazil). Every root canal was prepared using a ProTaper Next (Dentsply Maillefer, Ballaigues, Switzerland) system with a rotation speed of 280 rpm; this system was coupled with an electric motor, and the torque was 2 N. The initial exploration was performed with PathFile 13-16-19 instruments (Dentsply Maillefer, Ballaigues, Switzerland). Next, the sequence of instruments X1, X2 and X3 was applied up to the working length, which was defined as 1 mm below the apical foramen. At each instrument change, 2 mL of irrigating solution was used; thus, a total of 12 mL of irrigant was used. Final irrigation with 17% EDTA was performed for 3 min in groups G2 and G3. In group G4, 12 mL of a mixture of 5% NaOCl with 18% HEBP was used for 25 min. For irrigation procedures, a NaviTip needle of 21 mm and 30 ga diameter (Ultradent Products Inc, South Jordan, UT, USA) was positioned 3 mm short of the working length. The root canals were irrigated for 25 min to simulate the biomechanical preparation time. After this step, all roots received a final irrigation with 10 mL of distilled water. The canals were dried with paper cones and were stored at 37^°^C at 100% moisture for 7 days until the resistance tests were performed. 

***Preparation for the mechanical test***

After preparation, the roots were covered with wax (Utility Wax Sheets, Kerr Dental, Orange, USA) to a level of 2 mm from the cervical edge to create a thickness of 0.2-0.3 mm of wax. Then, the roots were vertically mounted in copper molds filled with autopolymerizing acrylic resin (JET - Clássico, SP, Brazil) in standard cylinders and adapted for the mechanical compression test in the Universal Testing Machine (Dynamometers KRATOS, LTDA, SP, Brazil). The samples were then polymerized for 24 h. After polymerization, the roots were removed from the mold and placed in hot water for two sec for wax removal. Polyether impression material with a heavy-bodied consistency (Impregum Soft, 3M ESPE, SP, Brazil) was proportioned and mixed with base pastes and catalyst following the manufacturer's instructions and inserted into the root-shaped space, which was then repositioned under digital pressure until the cervical boundary was marked. Excess polyether was removed with a scalpel blade (Solidor - Lamedid, Osasco, SP, Brazil). This initial procedure was aimed at replicating the periodontal ligament with a thickness of 0.2-0.3 mm as previously described in the literature [19]. 

The cylinders were then mounted on the base of the Kratos machine. The 4-mm diameter spherical stainless-steel tip was directed parallel to the long axis of the tooth and centered over the root canal orifice. The apparatus was calibrated to operate the disseminator using a vertically compressive load in the root canal at a speed of 1.0 mm/min with 50 cm of maximum displacement and a force of 600 N. The load was vertically applied along the longitudinal axis of the tooth. The program automatically recorded the maximum force applied to the root by the disseminator to produce a canal fracture. The fracture was defined as the point where the force decreased and the sound emitted by the fracture was heard. The load at fracture was recorded in Newton’s (N). 

**Table 1:** Division of groups according to irrigation regimes (*n*=10

**Group**	**Initial irrigation (25 min)**	**Final irrigation (3 min)**
**G1**	0.9% NaCl	
**G2**	2.5% NaOCl	17% EDTA
**G3**	2% CHX gel	17% EDTA
**G4**	1:1 mixture of 5% NaOCl + 18% HEBP	

**Table 2:** The mean (SD), maximum and minimum values of fracture resistance of teeth in Newton’s (N

**Irrigation regime**	**Mean (SD)**	**Minimum**	**Maximum**
**0.9% NaCl**	1051.42^a^ (459.62)	548.97	2095.36
**2.5% NaOCl + 17% EDTA**	822.21^a^ (276.63)	333.47	1160.97
**2% CHX gel + 17% EDTA**	981.10^a^ (306.64)	617.32	1602.73
**1:1 mixture of 5% NaOCl + 18% HEBP**	623.88^b^ (158.19)	410.07	882.10

* *Different letters in the same column represent significant differences, P<0.05*

***Statistical analysis***

The data were assessed for normality using the Shapiro-Wilk test (*P*>0.05). The normality values assumed in each group were as follows: G1 (*P*=0.056), G2 (*P*=0.559), G3 (*P*=0.371) and G4 (*P*=0.400). To compare the means of the sample groups with respect to the RFR, analysis of variance (one-way ANOVA) and a subsequent multiple comparison test (Tukey’s test) were used with a confidence level of 95%.

## Results

The mean fracture resistances of the test and control groups are shown in Table 2. Group G1 (0.9% NaCl) showed greater fracture resistance when compared to groups G2, G3 and G4 (*P*>0.05). No significant differences were found between group G2 (2.5% NaOCl+17% EDTA) or group G3 (2% CHX gel+17% EDTA) and the control group G1 (*P*>0.05). A significant difference was identified for group G4 (mixture of 5% NaOCl+18% HEBP) (*P*<0.05).

## Discussion

Shaping during instrumentation of the root canal, preparation for rehabilitation with intraradicular pinus and loss of moisture caused by the absence of pulp tissue are factors that modify the mechanical integrity of endodontically treated teeth and consequently reduce the RFR [20]. In this context, it was observed that the deleterious effects caused by irrigation solutions include dentin dehydration [21] and altered dentin microhardness [12], flexural strength and modulus of elasticity [22] as well as the erosion of dentin tissue [4, 11, 23] and the oxidation of organic components [1]. Although these studies demonstrate the effect of irrigating solutions on the mechanical properties of root dentin, in the clinical scope, these results are extrapolated because of the greater susceptibility to fracture of treated teeth and by interfering with tooth longevity [7, 22]. Thus, investigations about the RFR are necessary after the use of conventional irrigation protocols.

Irrigation solutions promote changes in the morphology and physical and chemical composition of dentin [4]. Therefore, for endodontic treatment, the search for the ideal irrigation regime aims to maintain the integrity of the mechanical properties of dentin tissue while promoting tissue dissolution and antimicrobial activity. Thus, the current study evaluated the effect of three irrigation regimes on RFR. According to the results, the highest mean RFR was seen in G1 (0.9% NaCl), followed by G3 (2% CHX gel+17% EDTA) and G2 (2.5% NaOCl+17% EDTA). However, the values were not significantly different. The lowest mean RFR was seen in G4 (5% NaOCl+18% HEBP) (*P*<0.05).

According to the present investigation, it can be affirmed that in comparison with the G1 group, all tested irrigation regimens presented lower mean values of RFR. However, the results obtained should be carefully analyzed when extrapolating them to clinical conditions. In this study, root canal obturation was not performed because clinicians are aware of the potential risk of damage during some root filling procedures. Several previous studies have demonstrated that various obturation and filling techniques can create fractures or cracks [24 - 26]. Barreto *et al.* [24] concluded that apical pressure filling techniques affect the occurrence of root fractures. Shemesh *et al. *[25] reported that the lateral compaction technique produces more cracks than the non-compaction filling technique, and Capar *et al. *[26] stated that filling techniques caused more cracks than the use of instrumentation alone.

Despite these results, the isolated action of different irrigation regimes with different chelating agents on RFR after instrumentation should be examined due to their deleterious effects on root dentin tissue and considering the possibility of root fracture between endodontic treatment sessions. Regardless of the irrigation agent used in G2 (2.5% NaOCl+17% EDTA) and G3 (2% CHX gel+17% EDTA), it was observed that the final use of EDTA as an irrigant did not decrease RFR. It has previously been reported that the effects of the interaction of mineralized dentin with NaOCl solution facilitate the penetration of the EDTA, which then leads to the dissolution of apatite. When used as a solvent for inorganic matter after preparation of the root canal with NaOCl, EDTA dissolves the “sparse ghost mineral layer” in collagen [1, 4, 27] and also exposes the underlying dentin, which is irreversibly destroyed by NaOCl. As a result, acids produced by NaOCl gradually dissolve the remaining apatite crystals on this layer [1]. In contrast with the present results, NaOCl action appeared not to significantly decrease RFR.

Zenhder *et al. *[18] suggested that in an alternating irrigating regimen, copious amounts of NaOCl should be administered to rinse out chelator remnants and to allow the NaOCl to develop its antimicrobial and tissue-dissolving potential [18]. In the current study, it is possible that the organic portion of dentin was not sufficiently affected by the proteolytic action of NaOCl because EDTA was the final irrigant and NaOCl was not used again; thus, the remaining smear layer probably influenced the RFR. These results can be related with previous findings regarding mineral content and the ultrastructure of root canal dentin showing that when used after EDTA, NaOCl acts directly on the collagen that is widely exposed by the demineralization by the chelating agent [4, 28]. In relation to the present results in G2 (2.5% NaOCl+17% EDTA), irrigation with 2.5% NaOCl after the use of 17% EDTA was not realized, which may have contributed to a smaller effect on the organic components of dentin.

In another previous study, Turk *et al.* [29] evaluated the effect of CHX on RFR with various concentrations of EDTA and concluded that CHX irrigation following EDTA/NaOCl irrigation increased RFR in teeth filled with AH Plus [29]. In our results, it is possible that CHX might have prevented the degeneration of collagen by matrix metalloproteinases (MMPs), thus causing the self-degradation of collagen in demineralized dentin [26, 30] and resulting in the lack of a significant difference in RFR values for the G3 group (2% CHX gel+17% EDTA).

The chelating action of EDTA causes alterations in the microstructure of dentin [4, 17, 31]. This process is responsible for dentin erosion due to the demineralization of inorganic components by the chelation of calcium ions present in the hydroxyapatite [4, 12]. In the current study, the results observed for the experimental groups suggest that the decreases in RFR in comparison with the control group are related to the use of chelating agents. This can be seen in the results for G4, where the use of a 5% NaOCl+18% HEBP mixture as the final irrigant in teeth resulted in lower resistance to fracture. 

These results most likely reflect the time-dependent characteristic of HEBP when used in a unique solution with NaOCl that maintained contact with dentin tissue for 25 min, thereby reducing the RFR. On the other hand, in groups G2 and G3, 17% EDTA was used as the final irrigant with a time of contact of 3 min and presented no significant differences in relation to group G1. In a previous study, Uzunoglu *et al.* [6] reported that the use of 17% EDTA decreased the fracture resistance of teeth more than that produced by 5% EDTA after 10 min of application; however, the results were reversed when the solutions were used for 1 min of application [6], and this finding has relevance for the present results.

In this study, a mixture of 5% NaOCl+18% HEBP was applied for 25 min in group G4, considering that the use of HEBP as a weak chelator has been proposed as an alternative to EDTA use and that the possibility exists to combine HEBP with NaOCl without losing the desired properties of either compound [4, 12, 13, 17, 18]. The decrease in RFR in G4 showed different results from those obtained in a recent study that evaluated the direct action of HEBP on the collagen fibers of dentin tissue [4]. The previous results suggested that HEBP may have led to a more superficial action of NaOCl on the organic portion of the dentin, as EDTA has a greater action on inorganic components [4, 13] in addition; higher levels of calcium and phosphorus minerals were found in root dentin tissues treated with HEBP [4]. The most probable explanation for these results is related to the time-dependent aspect of HEBP. When 17% EDTA is used, it has been suggested that the action of a final rinse for only 3 min is limited to the smear layer. In this experiment, this solution probably did not reach the underlying dentin tissue; thus, 17% EDTA may have led to a more superficial action while HEBP maintained contact with the dentin tissue for longer than EDTA and directly affected the RFR.

Due to the *in vitro* nature of this experiment, the use of standardized specimens and conditions for use in the mechanical tests restricts the other causes that may be related to root fracture such as the design of nickel-titanium rotary instruments and the presence of parafunctional habits [32, 33]. It is important for dentists to understand that root fracture is a multifactorial problem and is related to the clinical condition of each patient. Therefore, more studies are necessary to clarify the influence of other irrigation regimes by varying the application time and concentration of irrigants and to determine its effects on dentin tissue and the relationship with root fracture.

## Conclusion

The combined solution of 5% NaOCl and 18% etidronate decreased root fracture resistance. Although etidronate is considered a weak chelator, this irrigant solution acts time dependently and directly interferes with root fracture resistance.
